# Alternative approach to the DART mission by the use of gravity assist maneuvers with the Moon and solar sails

**DOI:** 10.1038/s41598-023-33680-4

**Published:** 2023-04-28

**Authors:** Rebeca S. Ribeiro, Lucas G. Meireles, Antônio F. B. A. Prado, Cristiano F. de Melo

**Affiliations:** 1 Postgraduate Division, National Institute for Space Research, São José dos Campos, Brazil; 2 Peoples’ Friendship University of Russia (RUDN), Moscow, Russian Federation; 3 Department of Mechanical Engineering, Federal University of Minas Gerais, Belo Horizonte, Brazil

**Keywords:** Aerospace engineering, Asteroids, comets and Kuiper belt

## Abstract

The formation of our Solar System and planetary defense strategies are among the priorities to be investigated in the next years by the space science community. As *in-situ* missions to small bodies (as comets and asteroids) are options to conduct these investigations, this paper proposes a combination of methodologies to produce low-cost transfers to near-earth asteroids (NEAs). Low-cost trajectories derived from retrograde periodic orbits around ${L}_{1}$ are taken as a starting point for the escape of the Earth-Moon system and, as the vehicle exits the sphere of influence of Earth, the deployment of an adjustable solar sail guarantees the interception of the target in a predetermined position and time of flight. Different sail loadings (164, 61 and 30 g/m^2^) are tested and a case study to the NEA 65,803 Didymos is presented. The results show economies in the velocity increments required by the mission up to 8.48%, although a longer time of flight might be needed depending on the sail loading.

## Introduction

Over the past years, the space science community has been committed to define the themes and key questions that will guide the planning of new space missions and investigations related to space, to better allocate the funds available for science development^1^. Among the priority topics raised by the European Space Agency (ESA) and the Planetary Science Division (PSD) of the National Aeronautics and Space Administration (NASA), for example, for this and the next decade, we highlight the question to better understanding the formation and evolution of the Solar System and the development of planetary defense strategies^2,3^. In common, these two topics have the study of small bodies of our system (as comets and asteroids), either by ground-based observations or with in situ missions, as sample return missions^2^.

Both NASA’s Double Asteroid Redirection Test (DART) mission and ESA’s HERA missions are efforts of these space agencies in answering the questions raised by the space science community. DART’s probe, launched in November 2021, had the goal of making a kinetic impact with the smallest body of the double asteroid 65803 Didymos, called Dimorphos^4^. The goal was to perform a technological test of methods for planetary defense and it was achieved in September 2022^4^, when, after the impact of the probe, the data collected by the mission team confirmed the alteration of the asteroid’s orbit^5^. HERA mission, on the other hand, has its launched scheduled for 2024 and has as a goal the characterization of the physical properties of this asteroid and the analysis of the effects of the impact of DART’s probe on it^6^. In the following years, at least two missions are scheduled for Near-Earth Objects (NEOs), specially for Near-Earth Asteroids (NEAs), NASA’s NEA Scout^7,8^ and NEO Surveyor^9,10^.

Given the prospect of further missions to NEOs, it is reasonable to study new methods and techniques that enable low-cost transfers to these celestial bodies. Thus, this work proposes a combination between swing-by maneuvers with the Moon, starting from periodic orbits around the Lagragian point *L*_1_ in the Earth-Moon system, and solar sails to, at the same time, increase the reach of a spacecraft that has a NEA as aim and decrease the required velocity increments ($\Delta V$_s_) to accomplish this transfer.

Swing-by maneuvers (or gravity assist maneuvers) consist of close approaches between a spacecraft and massive bodies, as planets or moons, such that the vehicle has its energy and angular *momentum* increased or decreased and, therefore, its orbit changed as a result of these encounters. As the high fuel consumption to reach targets far from Earth would make some missions impossible, several missions used swing-by maneuvers to enable deep space investigations. For example, NASA’s ISEE3/ICE performed five complex swing-bys with the Moon before starting its journey towards a comet^11^. Studies with general approaches on this type of maneuver can be found in Lawden^12^, Minovitch^13^ and Broucke^14^, while an interesting historical review of this technique can be found in the work by Negri and Prado^15^.

Complementary to swing-by maneuvers, periodic orbits of family G^16^ have been used to produce low-cost escape trajectories in the Earth-Moon system. The periodic orbits of family G were first introduced by Broucke^16^ for the Restricted Three Body Problem (R3BP), but its existence in more complex dynamics have already been documented in several works^17–20^. For example, in de Melo, Macau and Winter^18^, as these orbits pass naturally close to the surface of both the Earth and the Moon, minimal variations in the velocity were introduced in the trajectory of the vehicle inside a periodic orbit of family G to cause a swing-by with the Moon and its escape from the Earth-Moon system. This strategy was used to plan missions for the NEAs 99942 Apophis, 1994WR12, and 2007 UW1. In more recent works^20^, different types of trajectories derived from periodic orbits of family G, called trajectories G (TGs), were mapped as function of the launch velocity, starting from a Low-Earth Orbit (LEO), and the position of all the bodies involved in the dynamics of the system. Ribeiro, de Melo and Prado^20^ demonstrated that the use of these TGs could lead to a decrease of 2.5–5% in the necessary $\Delta V$_s_ to perform transfers to NEAs, when compared to reference methods as the method of Lambert. In "Restricted four-body problem and trajectories G" Section, more details about these trajectories are presented.

Solar sails, on the other hand, make use of thin reflective membranes to continually accelerate a spacecraft under the influence of Solar Radiation Pressure (SRP)^21^. Just a few missions up to now were successful in deploying solar sails, but they showed promising results. As an example, we can highlight the IKAROS mission from the Japan Aerospace Exploration Agency (JAXA) that was launched in May 2010 and, after its success in becoming the first solar interplanetary solar sail, it turned into a stepping stone for the plans of the agency of a larger solar sail to visit the Trojan asteroids of Jupiter, to be launched in 2050^22^. After the failure of Cosmos 1 mission, the Planetary Society kept the development of solar sails projects until the successful launches of LightSail 1 and LightSail 2 missions, in 2015 and 2019, respectively^23^. While LightSail 1 mission objectives were restricted to functionality tests of the CubeSat platform and solar sail, LightSail 2 mission was able to demonstrate the use of propelled sailing by controlled orientation of the sail^23^. Furthermore, NEA Scout mission, previously mentioned, was recently launched, on November 16, 2022, as a secondary payload in the Artemis 1 mission^24^. The goal of the mission was to utilize a 6U CubeSat platform and a sail of 86 m^2^ to observe a NEA^8^, but communication with the spacecraft has been lost and the NEA Scout team is still unable to confirm the status of the spacecraft and its sail deployment^25^.

All aspects considered, in the dynamics of the Restricted Four-Body Sun-Earth-Moon-spacecraft Problem (R4BP), this paper presents an investigation of a strategy to reach NEAs that uses an escape TG (TGE), starting from a LEO, and deploys a solar sail at the moment the vehicle leaves the sphere of influence (SOI) of the Earth. The goal is to demonstrate the reduction of the required $\Delta V$_s_ for this transfer, when compared to reference methods. For that, a case study considering the NEA 65803 Didymos is presented.

The presentation of this paper follows the order: in "Restricted four-body problem and trajectories G" and "Solar sails" Sections, the theoretical and mathematical principles of TGs and solar sails are described, respectively. "Methodology" Section presents the methodology and the heuristic employed in this paper. "Applications" Section includes applications, in which a general example, in the R4BP, of the combination between the techniques previously mentioned is demonstrated in "Transfer in the restricted four-body sun-earth-moon-spacecraft problem" Section and the application of the method to NEA 65,803 Didymos is presented in "Transfer to 65803 Didymos" Section.

## Restricted four-body problem and trajectories G

For the Restricted Four-Body Problem that conducted this investigation, the dynamic system is composed by the Sun, Earth, Moon, and a spacecraft (SC) of negligible mass, henceforth associated with the indexes 1 through 4, respectively. The same normalization of mass, distance, and time parameters, usual for the R3BP Sun-Earth-Moon^26^, is also used so ${\mu }_{1}={\mu }_{1}/({\mu }_{2} + {\mu }_{3} ), {\mu }_{2}={\mu }_{2}/({\mu }_{2} + {\mu }_{3} ), {\mu }_{3}={\mu }_{3}/({\mu }_{2} + {\mu }_{3} ),$ and ${\mu }_{4}={\mu }_{4}/({\mu }_{2} + {\mu }_{3})$ are the gravitational parameters, with ${\mu }_{2}+{\mu }_{3}=1$. The distance between Earth and Moon (384,400 km) is taken as the unit distance, as the orbital period of the primaries (*T* = 27.321661 days) is taken to be 2$\pi$. The differentials equations of motion for the four bodies moving under mutual gravitational attraction forces are presented in Eq. (1) for an inertial (X, Y, Z) reference frame.

1 $$\ddot{{{\varvec{R}}}_{i}}=\sum_{\begin{array}{c}j=1\\ j\ne i\end{array}}^{4}\frac{{\mu }_{j}}{{R}_{ji}^{3}}\left({{\varvec{R}}}_{j}-{{\varvec{R}}}_{i}\right)$$

In Eq. (1), ${{\varvec{R}}}_{{\varvec{i}}}=({X}_{i},{Y}_{i},{Z}_{i})$ is the vector position of the i-th body in the reference frame, while ${R}_{ij}=\left|{{R}_{j}-R}_{i}\right|={\left[{\left({X}_{j}-{X}_{i}\right)}^{2}+{\left({Y}_{j}-{Y}_{i}\right)}^{2}+{\left({Z}_{j}-{Z}_{i}\right)}^{2}\right]}^\frac{1}{2}$, with $j\ne i$, is the distance between the i-th and j-th bodies, and ${\ddot{{\varvec{R}}}}_{{\varvec{i}}}$ is the acceleration of the i-th body.

**Figure 1:**
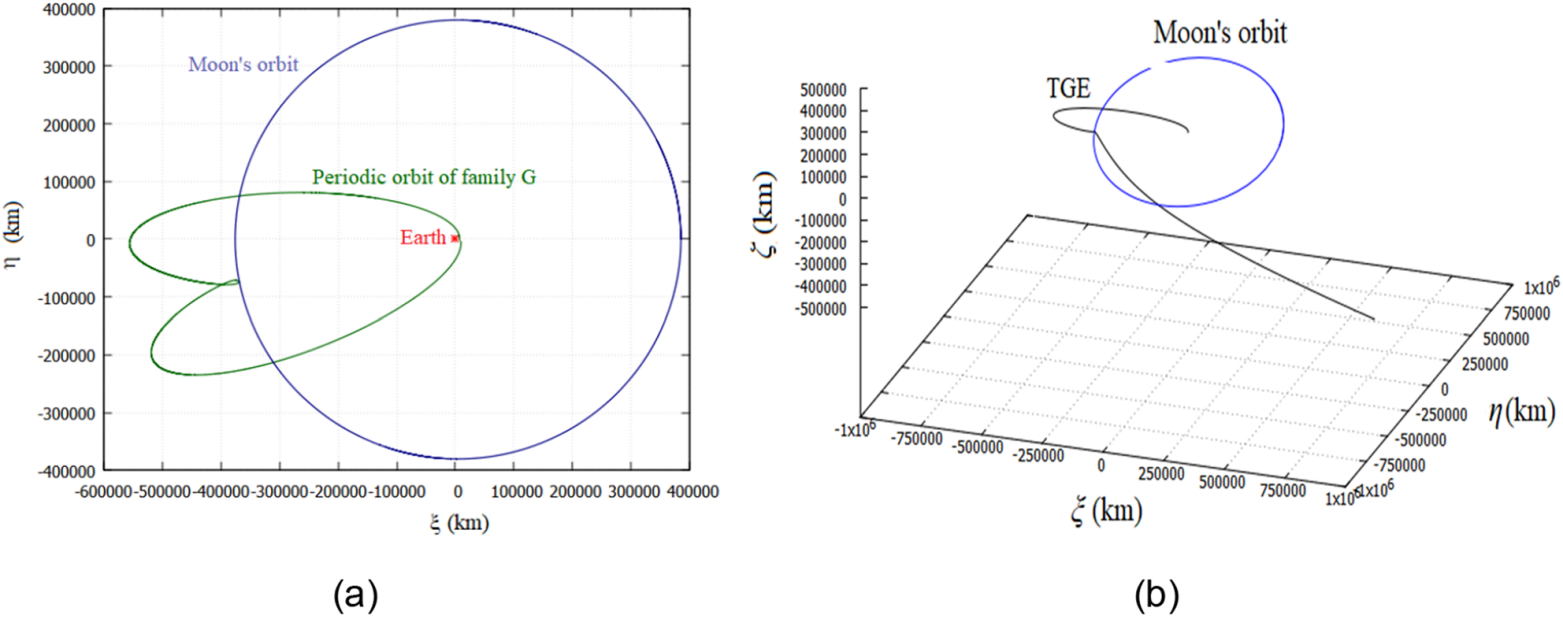
(**a**) Periodic orbit of family G and (**b**) trajectory G of escape in the inertial geocentric reference frame.

**Figure 2:**
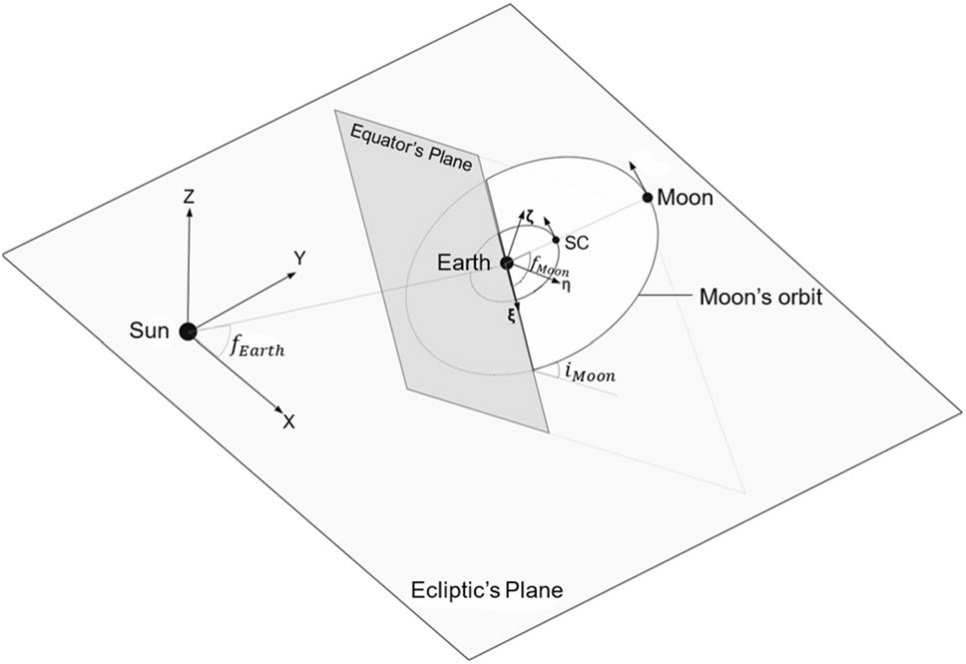
Relative positioning in the R4BP.

Low-cost escape trajectories are found in this system for a variety of initial conditions of position and velocity^20^. Starting from a circular LEO and the initial conditions for a periodic orbits of family G^16^, as the one showed in Fig. 1a in an inertial geocentric reference frame ($\xi , \eta ,\zeta$), as defined by Ribeiro, de Melo e Prado^20^, small increments of velocity are introduced in order to change the trajectory of the vehicle and to provoke a swing-by with the Moon, in what was called “trajectories G of escape” (TGE). The required injection velocity to produce trajectories G similar to the one shown in Fig. 1b is given by Eq. (2)^20^ as a function of the altitude of the initial parking LEO (${h}_{0}$), the longitude of the ascending node of the Moon (${\Omega }_{Moon}$), and the true anomaly of the Moon ($0^\circ <{f}_{Moon}<360^\circ$). A scheme showing these variables is presented in Fig. 2.

2 $${V}_{SC}=-8.481880\times {10}^{-4}{h}_{0}+\mathrm{11,103.421}+{\vartheta }_{4B}+Asin\left[B\left({\Omega }_{Moon}+{f}_{Moon}\right)+C\right]$$

**Figure 3:**
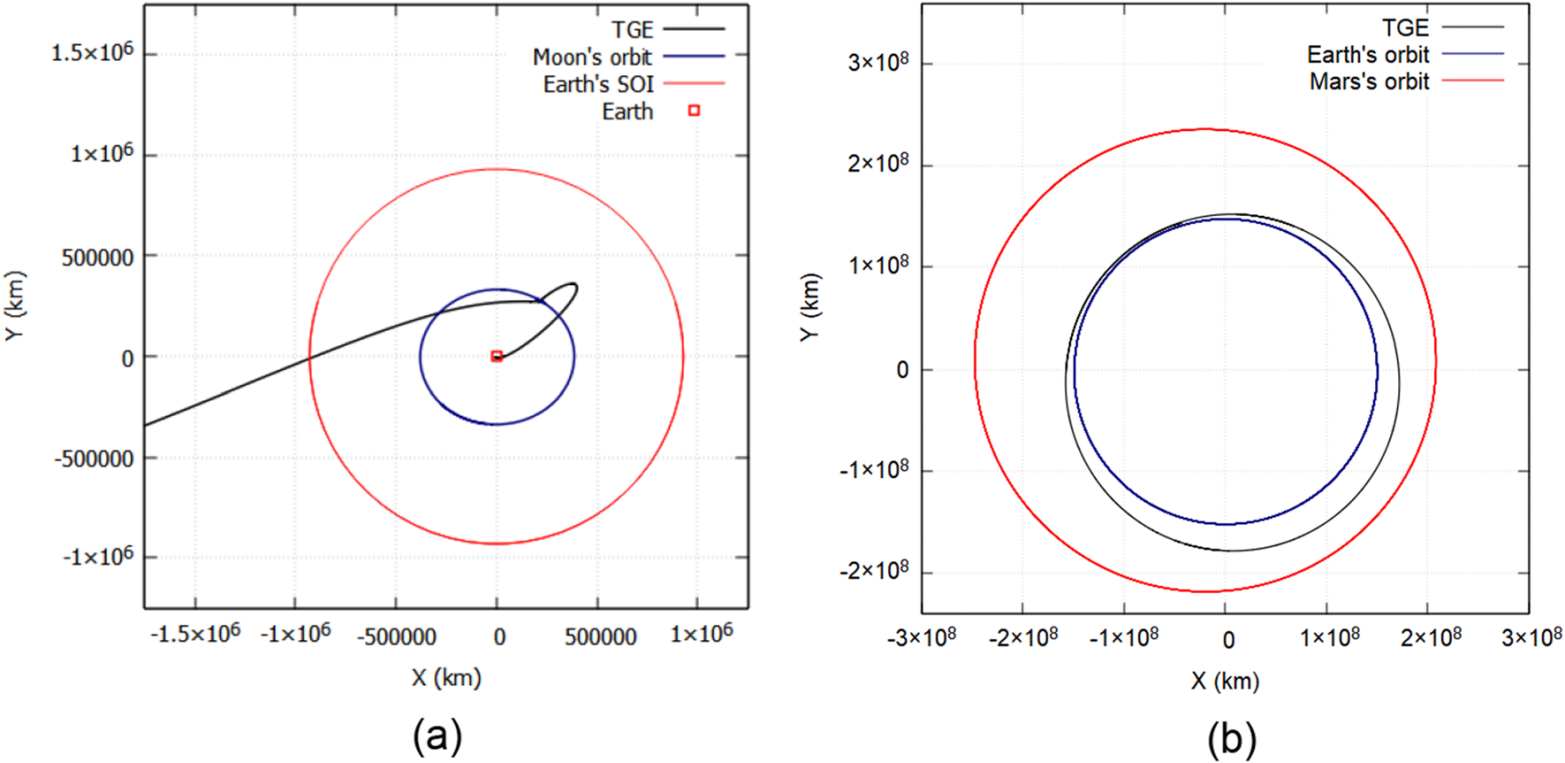
(**a**) TGE with maximum reach in the geocentric reference frame. (**b**) The same TGE in the heliocentric reference frame.

The trajectories G of escape provide low-cost alternatives to escape the Earth-Moon system towards Venus or Mars and can be generated when 0.312 $\pm$ 0.008 < ${\vartheta }_{4B}\le$ 0.667 $\pm$ 0.021 m/s in Eq. (2)^20^. The farthest reach TGE towards Mars has aphelion of 1.21 AU and perihelion around 1 au, while the farthest reach TGE towards Venus has perihelion of 0.82 AU and aphelion around 1 au. For example, a TGE with maximum reach towards Mars can be found for the following initial conditions^20^: ${h}_{LEO}=200 \mathrm{km},$ ${\Omega }_{Moon}=0$, ${f}_{Moon}=225.0^\circ$, so ${V}_{SC}=10.936093$ km/s. This TGE is presented in Fig. 3b in an inertial geocentric reference frame (X,Y,Z). After its escape from the Earth-Moon system, it has an aphelion radius ${R}_{ext}$ = 181,272,593.0 km (1.21 AU).

Given the characteristic velocity of a 200 km LEO, ${V}_{c}=7.788440$ km/s, the total $\Delta V$ for this transfer via TGE is $\Delta {V}_{TGE}={V}_{SC}-{V}_{c}=3.151755\mathrm{ km}/\mathrm{s}$, while the first $\Delta V$ for a transfer via the patched conics method, starting from the same LEO to ${R}_{ext}$, is 3.313827 km/s. Therefore, this transfer via TGE by itself represents a reduction of 4.89% in the total $\Delta V$.

## Solar sails

**Figure 4:**
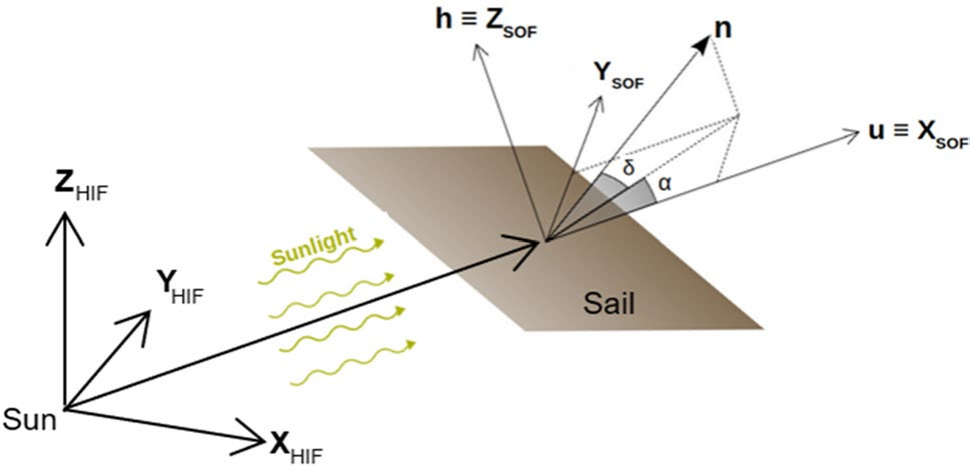
HIF e SOF.

To calculate the acceleration experienced by the spacecraft given by a solar sail, the model presented by Vulpetti et al.^27^ is used. The acceleration is initially defined in a fixed coordinate system centered in the center of mass of the spacecraft (SOF) and later transformed to a heliocentric inertial frame (HIF) to describe the movement of the vehicle. Figure 4 shows a scheme of both reference frames.

According to Vulpetti et al.^27^, the x-axis of SOF is determined by the unit vector ${\varvec{r}}={\varvec{R}}/R$, where **R** is the vector position of the spacecraft in the HIF and R its module. The z-axis has the same direction of the spacecraft specific angular momentum (${\varvec{h}}$). However, if the spacecraft is in a retrograde orbit, the z-axis is oriented opposite to ${\varvec{h}}$. Finally, the y-axis completes the right-handed frame.

The acceleration in SOF is given by

3 $${\varvec{A}}^{{{\varvec{SOF}}}} = g_{ \odot } {\mathbf{L}},$$

where

4 $$g_{ \odot } = \left( {\frac{{GM_{ \odot } }}{{R^{2} }}} \right)$$

is the magnitude of the solar gravitational acceleration at a distance R, expressed in astronomical units, in the SOF, G is the gravitational constant and ${M}_{\odot }$ is the mass of the Sun. While, also in SOF,

5 $$\mathbf{L}=\left(\frac{1}{2}\frac{{\sigma }_{C}}{\sigma }\right){n}_{x}\left[\left(2{r}_{spec}{n}_{x}+{\chi }_{f}{r}_{diff}+\kappa a\right)\mathbf{n}+\left(a+{r}_{diff}\right)\mathbf{u}\right]$$

is the lightness vector, ${\varvec{L}}=({l}_{x}, {l}_{y}, {l}_{z})$, a dimensionless vector defined by physical properties of the solar sail, such as the sail loading ($\sigma$), critical sail loading (${\sigma }_{C}$), specular reflectance coefficient (${r}_{spec}$), diffuse reflectance coefficient (${r}_{diff}$), coefficient of emissive/diffuse momentum ($\chi$), and absorptance coefficient ($a$). $\kappa$ is a dimensionless factor defined by Eq. (6)^27^, where $\varepsilon$ denotes the temperature-dependent emittance of the surface and *T* is the sail temperature. The subscripts $f$ and $b$ refer to the front and back of the sail, respectively.

6 $$\kappa \equiv \frac{{\chi }_{f}{\varepsilon }_{f}\left(T\right)-{\chi }_{b}{\varepsilon }_{b}\left(T\right)}{{\varepsilon }_{f}\left(T\right)+{\varepsilon }_{b}\left(T\right)}$$

In Eq. (5), ${\varvec{n}}=({n}_{x},{n}_{y},{n}_{z})$ is a unit vector orthogonal to the sail defined in SOF, as ${\varvec{u}}$, also in SOF, is defined by ${\varvec{u}}=(\mathrm{1,0},0)$, according to Fig. 4. The orientation of the **n** vector and, consequently, the orientation of the sail, is defined by two angles in the SOF: the azimuth (α) and elevation (δ). The first is the angle between the XY components of **n** and the direction of the incoming sunlight **u**. The second is the angle between **n** and its XY components.

The acceleration in HIF is then given by the transformation equation

7 $${\mathbf{A}}^{\mathrm{HIF}}=\Xi {\mathbf{A}}^{\mathrm{SOF}},$$

where Ξ is an orthogonal transformation matrix defined by

8 $${\Xi } = \left( {{\mathbf{r}} {\mathbf{h}} \times {\mathbf{r}} {\mathbf{h}}} \right).$$

## Methodology

A spacecraft, initially placed in a 200 km circular parking LEO, receives a $\Delta {V}_{TGE}$ injection to be launched in a TGE, at $t=0$. Once in this trajectory, at the moment the vehicle reaches the limit of Earth's SOI, the region highlighted in red in Fig. 3a, the solar sail is deployed. The deployment mechanism is not covered by this investigation.

A great advantage of using a solar sail spacecraft after lunar fly-by maneuvers is that the solar sail is able to reorient itself in order to reach the same predetermined target, despite having small variation in its initial conditions. This means that solar sails are able to compensate small deviations from nominal trajectories, considering the uncertainties coming from the gravity assist maneuvers introduce in the planning of space missions.

Different values for the sail loading (σ) are considered for the transfer towards a target asteroid. Considering the vehicle of the NEA Scout mission as a reference (a 14 kg spacecraft with a solar sail of 85 m^2^)^7^, a $\sigma$ of 164.7 g/m^2^ is firstly used in the simulation. Other values for the loading are then tested: 61.2 and 30.6 g/m^2^, in which the latter is a theoretical threshold regarding the future development of solar sail technology^28^.

To guide the spacecraft in a collision course with the asteroid, it is needed to define the azimuth (α) and elevation (δ) angles of the sail as a function of time, which in turn, defines the resulting solar radiation pressure acceleration magnitude and direction throughout the simulation. The heuristic technique employed to define *α*(*t*) and *δ*(*t*) is taken from Meireles et al.^29^. In summary, it can be broken down into a series of steps, such as at each step (t) of the numeric integration:

- The spacecraft position at instant ${t}_{F}$, ${{\varvec{r}}}_{SC/HIF}({t}_{F})$, which is the desired collision instant, is determined in the HIF. This final position projection, which is updated at each instant *t*, is made considering the spacecraft osculating orbital elements at *t* and a null SRP acceleration from *t* to ${t}_{F}$. This is done considering that the spacecraft is under action only of the gravitational attraction of the Sun and by solving Kepler's equation of an elliptical orbit.
- A “Final Position Oriented Frame” (FPOF) is defined from ${\mathbf{r}}_{SC/HIF}({t}_{F})$. The position of the Sun is its origin and the X_FPOF_-axis points in the direction of the position of the spacecraft at ${t}_{F}$, ${\mathbf{r}}_{SC/HIF}({t}_{F})$. Its fundamental plane corresponds to the orbital plane of the spacecraft osculating orbit or, in other words, the Z_FPOF_-axis points in the direction of the spacecraft angular momentum at ${t}_{F}.$ Finally, the Y_FPOF_-axis is obtained by completing the right-hand rule system.
- A set of three properties, called guidance properties, are taken from the spacecraft final position projection (${{\varvec{r}}}_{SC/FPOF}({t}_{F})$) and the target position, which is the position of the asteroid at tF (${{\varvec{r}}}_{AST/FPOF}({t}_{F})$), both represented in the FPOF:
  - (P1): Angle between ${{\varvec{r}}}_{SC/FPOF}({t}_{F})$ and $pro{j}_{{X}_{FPOF} {Y}_{FPOF}}$(${{\varvec{r}}}_{AST/FPOF}({t}_{F}))$ (the projection of the target position onto the XY plane). To deal with the same units for all the properties (meters), P1 is multiplied by a normalization factor equal to 1 AU. In this manner, it represents the arc of a circle with a radius equal to 1 AU.
  - (P2) Difference between the magnitude of ${{\varvec{r}}}_{SC/FPOF}({t}_{F})$ and ${{\varvec{r}}}_{AST/FPOF}({t}_{F})$;
  - (P3) Z_FPOF_-axis component of ${{\varvec{r}}}_{AST/FPOF}({t}_{F})$.
- The algorithm determines the values of α(*t*) and δ(*t*), based on the values of P1(*t*), P2(*t*) and P3(*t*). The magnitudes of α(*t*) and δ(*t*) require further explanation, which can be taken from Meireles et al.^29^. They are a consequence of the rate of correction of P1, P2 and P3. Nevertheless, in summary:
  - If (P1(*t*) > 0) or (P2(*t*) < 0): α(*t*) > 0°.
  - If (P1(*t*) < 0) or (P2(*t*) > 0): α(*t*) < 0°.
  - The value of δ(*t*) depends on the value of P3(*t*) and the position of the spacecraft relative to the target.

To achieve a collision, the objective is to obtain P1(${t}_{F}$) = P2(${t}_{F}$) = P3(${t}_{F}$) = 0.

This methodology used to determine the sail orientation differs from conventional methods as the ones presented by Peloni et al.^30^ and Mengali et al.^31^ by presenting a fast solution for the search of interception trajectories to a target position in space. The technique is applied to searches with restraints to the final position of the spacecraft with a fixed transfer time, while focusing on maintaining a fixed sail orientation through different stages of operation within the mission. In turn, this reduces the number of attitude maneuvers necessary throughout a mission.

## Applications

This section is divided into two parts. In the first of them, a general example for the R4BP is presented to demonstrate the procedures implemented in this paper. In the second one, multiple missions to the NEA Didymos are considered, with different sail loading and launch dates.

### Transfer in the restricted four-body sun-earth-moon-spacecraft problem

Following the methodology presented in "Methodology" Section, a vehicle is launched in the farthest reach TGE, described in "Restricted four-body problem and trajectories G" Section, and deploys its solar sail after approximately 22 days from the start of the simulation, when it crosses the Earth’s SOI. For this general example, only a load of $\sigma = 164.6\;{\text{g}}/{\text{m}}^{2}$ was employed in the investigation.

**Figure 5:**
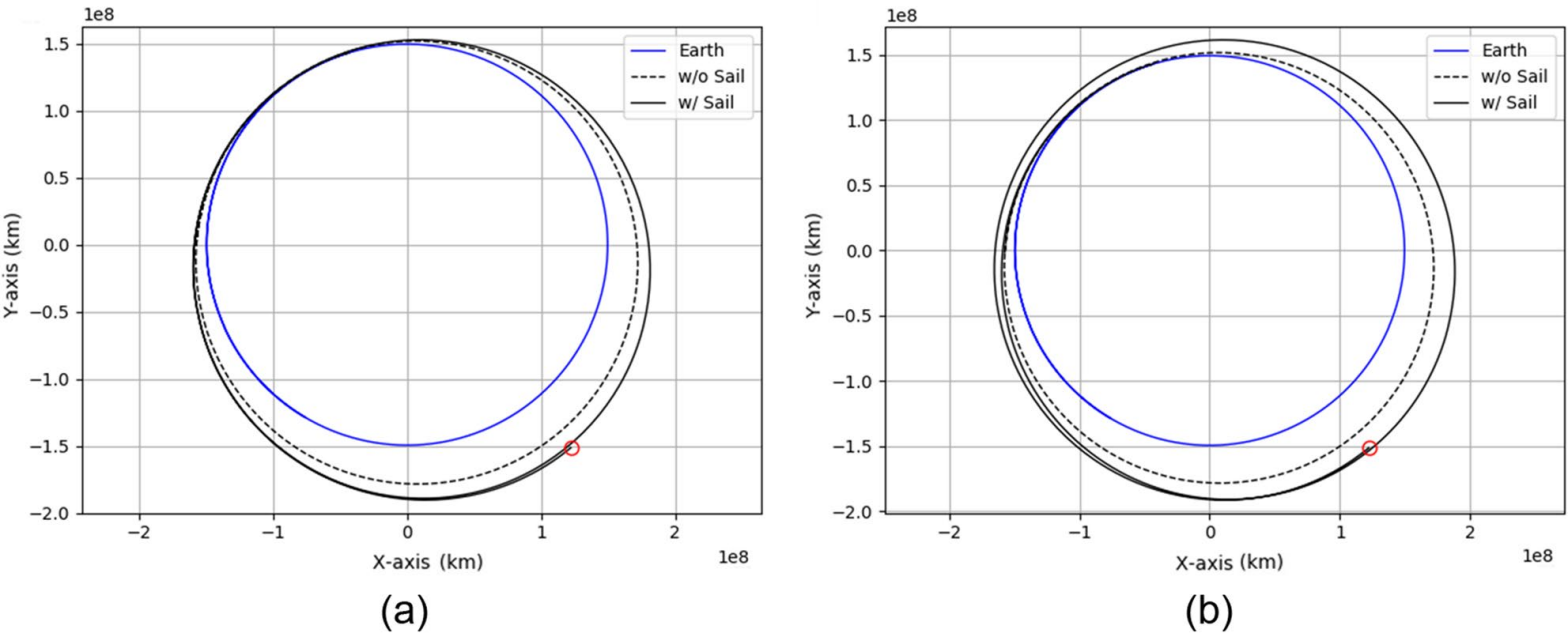
Heliocentric trajectories for the spacecraft with and without sail. (**a**) Tof of 700 days. (**b**) Tof of 725 days.

The method to adjust the solar sail is applied to perform a maneuver similar to an interplanetary Hohmann transfer, i.e., with a 180° transfer angle between the exit of Earth’s SOI and the region circled in red in Fig. 5, which shows the heliocentric trajectories of the spacecraft with and without the sail for times of flight (tof) of 700 and 725 days.

This section serves as an initial presentation of the application of TGEs with solar sails. Given the arbitrary choice of the final position and the sail loading considered for this initial demonstration, only a set of TGEs offered the possibility of a successful interception. This is because, given the low-thrust nature of solar sails, the shorter the time of flight and the higher the sail loading, the less a solar sail can redirect the spacecraft into an interception course. The TGE considered was chosen from this set as the first success case of interception.

**Figure 6:**
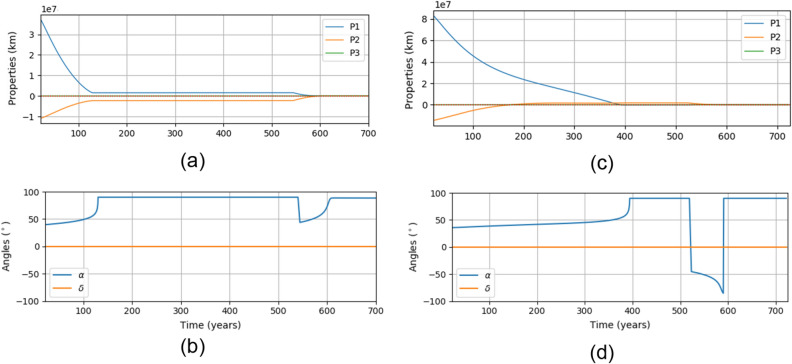
Guidance properties and orientation angles $\alpha$ and $\delta$. (**a**) Properties for transfer with tof of 700 days. (**b**) Angles for transfer with tof of 700 days. (**c**) Properties for transfer with tof of 725 days. (**d**) Angles for transfer with tof of 725 days.

The angles $\alpha$ and $\delta$ of the sail were adjusted as shown in Fig. 6b and c to guarantee that the spacecraft would reach the predetermined target position. As no changes in the plane of the orbits were necessary, $\delta$ remained zero throughout the entire path of the vehicle, while more abrupt variations in $\alpha$ were needed, especially for the 725 days transfer, as seen in Fig. 6d. Figure 6a and c present the changes in properties P1, P2, and P3 and, as they converge to zero, so does the solution regarding an interception with the target region.

At the end of the transfer, the vehicle with sail reaches an orbit with an aphelion of $194.47\times {10}^{6}$ km (1.29 AU), this distance is 7% greater than the one achieved without the sail. Such transfer, via the patched conics method^32^, would need a $\Delta V=3.385871 \mathrm{km}/\mathrm{s}$, therefore this method provides a $6.92\%$ reduction, as the only speed increment performed is $\Delta {V}_{TGE}=3.151755\mathrm{ km}/\mathrm{s}$, as stated in "Restricted four-body problem and trajectories G" Section.

These results show that a solar sail spacecraft is able to expand the launch window of a mission with no additional fuel expenditure, given the propellant-less nature of these space vehicles. This is a direct conclusion from the fact that, even with the same initial conditions and the same target, the spacecraft is able to reach it with different times of flight. Alternatively, by taking advantage of this same technique, the solar sail spacecraft would be able to reach the same target with the same transfer time, even with different initial conditions.

### Transfer to 65803 Didymos

In this section, transfers to NEA 65803 Didymos are presented. In each subsection a different sail loading is considered and in "Fuel saving and further discussions" Section a discussion is made about the possible impacts of the savings provided using the proposed method.

#### Sail loading of 30.6 g/m^2^

As the probe of the DART mission was launched on November 24, 2021, this date was set as a starting point for the search of TGEs that could be used in the planning of a mission aimed at Didymos. The ephemerids of the Earth, the Moon, and the NEA were collected in the JPL Horizons System^33^ and used in the investigation that found a suitable TGE for the transfer with $V_{SC} = 10.931883\;{\text{km}}/{\text{s}}$, and thus a $\Delta V_{TGE} = 3.143443\;{\text{km}}/{\text{s}}$.

**Figure 7:**
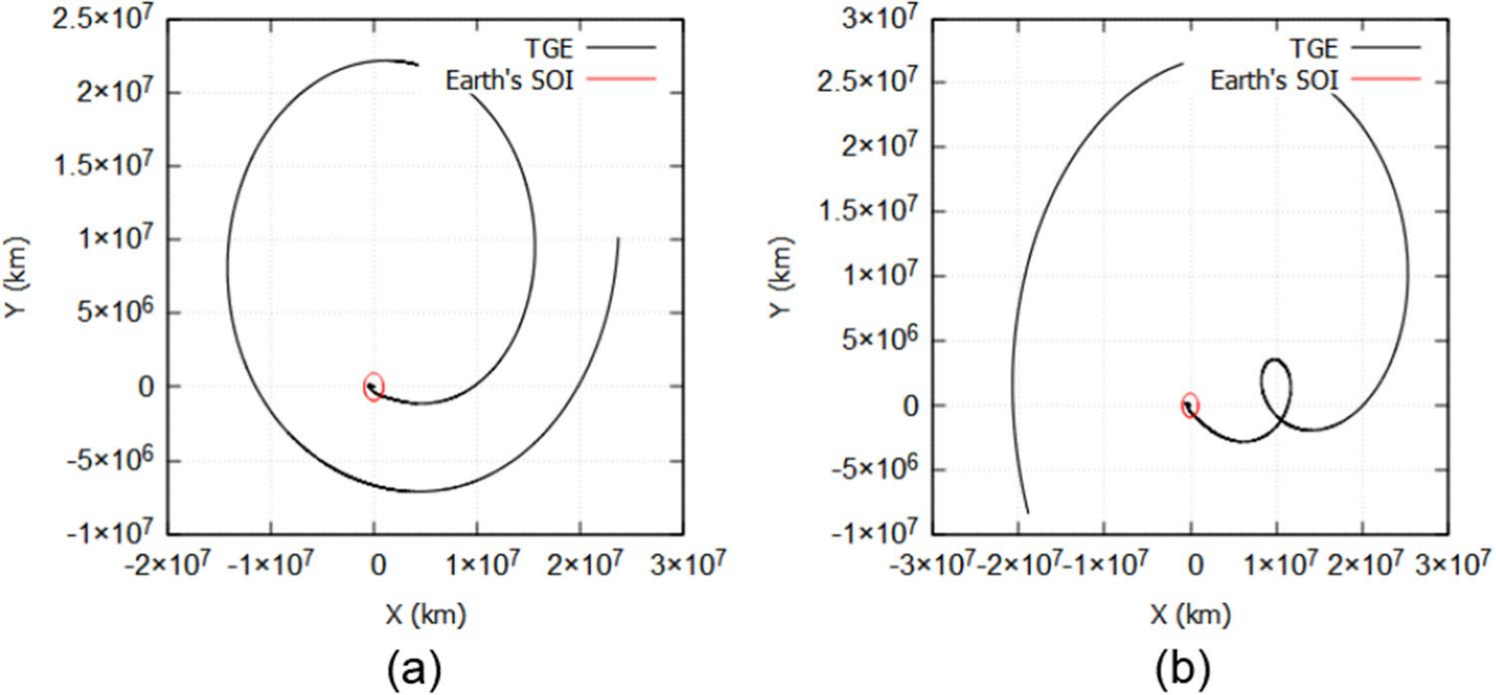
Examples of TGEs considered in the study, in the geocentric reference frame. (**a**) A direct and more feasible TGE to accomplish the transfer. (**b**) A more complex and not suitable TGE.

In this work, a feasible TGE is a trajectory that has a “direct” escape from the Earth-Moon system. Figure 7 presents two different TGEs in the geocentric reference frame to illustrate the screening process. While in Fig. 7a a TGE with a direct escape is presented, i.e., a possible candidate for the study, Fig. 7b presents a TGE that has a more complex path, which complicates the operation of the sail. Moreover, for these transfers to Didymos, TGEs with an escape in the direction of Venus, i.e., with the heliocentric semimajor axis less than 1 AU, proved to be more effective for the proposed methodology.

**Figure 8:**
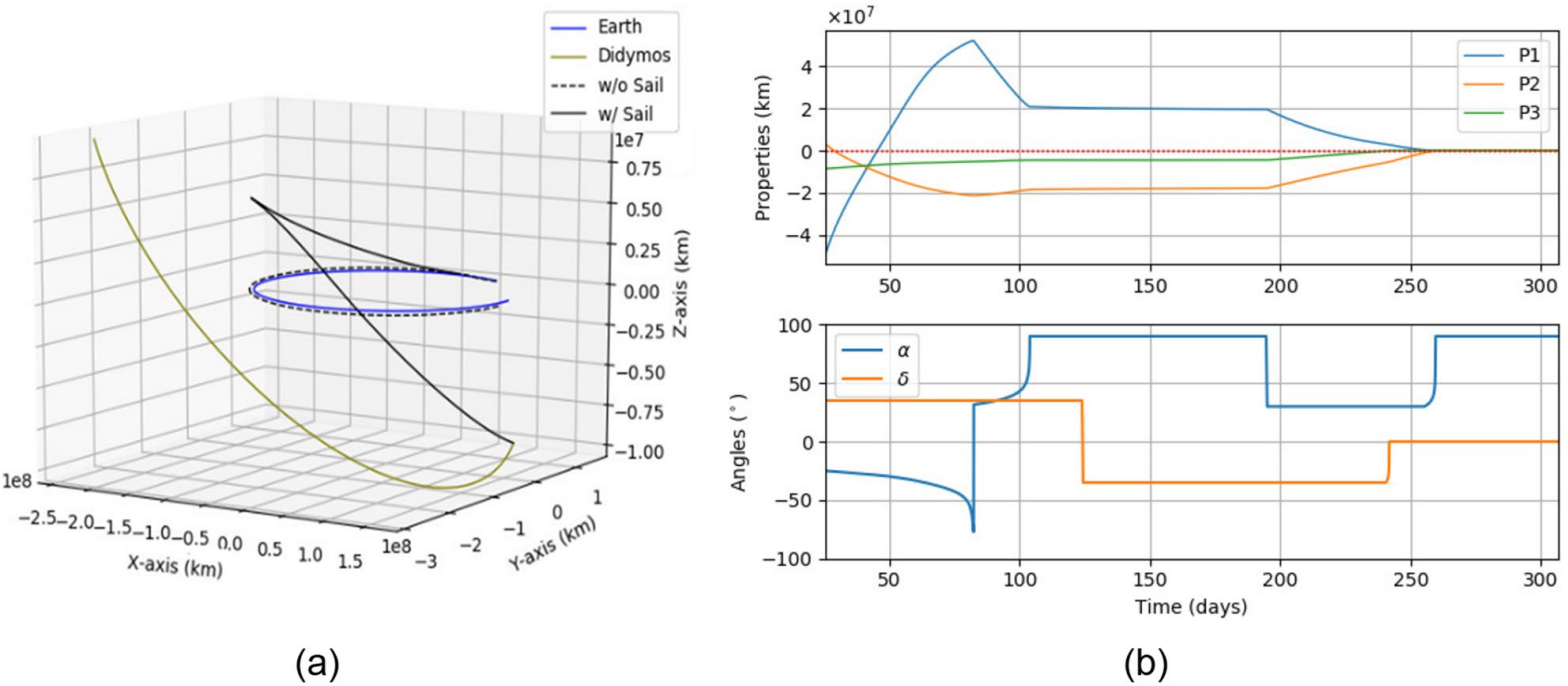
(**a**) Didymos interception for a tof of 306 days and sail loading of $30.6\;{\text{g}}/{\text{m}}^{2}$. (**b**) Sail guidance properties as a function of the time.

Once the TGE has been chosen, the same transfer procedure described in the "Transfer in the restricted four-body sun-earth-moon-spacecraft problem" Section is performed. Figure 8a shows the interception trajectory of the spacecraft and the NEA for a simulated tof of 306 days, for a load of $\sigma = 30.6\;{\text{g}}/{\text{m}}^{2}$. Figure 8b, in turns, shows the variation of properties as a function of the time during the transfer, such that P1, P2, and P3 had the final values of − 1.6, + 1.9, and + 2.6 m, respectively.

The interception date was set as September 26, 2022, for this to occur on the same day of the impact of the DART probe, therefore for a tof of 306 days. Using the method of Lambert^32^ to calculate the same interception, a $\Delta V_{L} = 3.434822\;{\text{km}}/{\text{s}}$ would be necessary. Thus, the $\Delta V_{TGE}$ represents a reduction of 8.48%.

It is important to note that similar TGEs offer similar solutions, from the perspective of the solar sail orientation. Small variations in the initial conditions (similar TGEs) create little difference in the solutions achieved. The choice for a particular TGE is based on a first success case, taken from a set of feasible TGEs for a given sail loading (as explained previously), considering the goal of intercepting the predetermined target.

**Figure 9:**
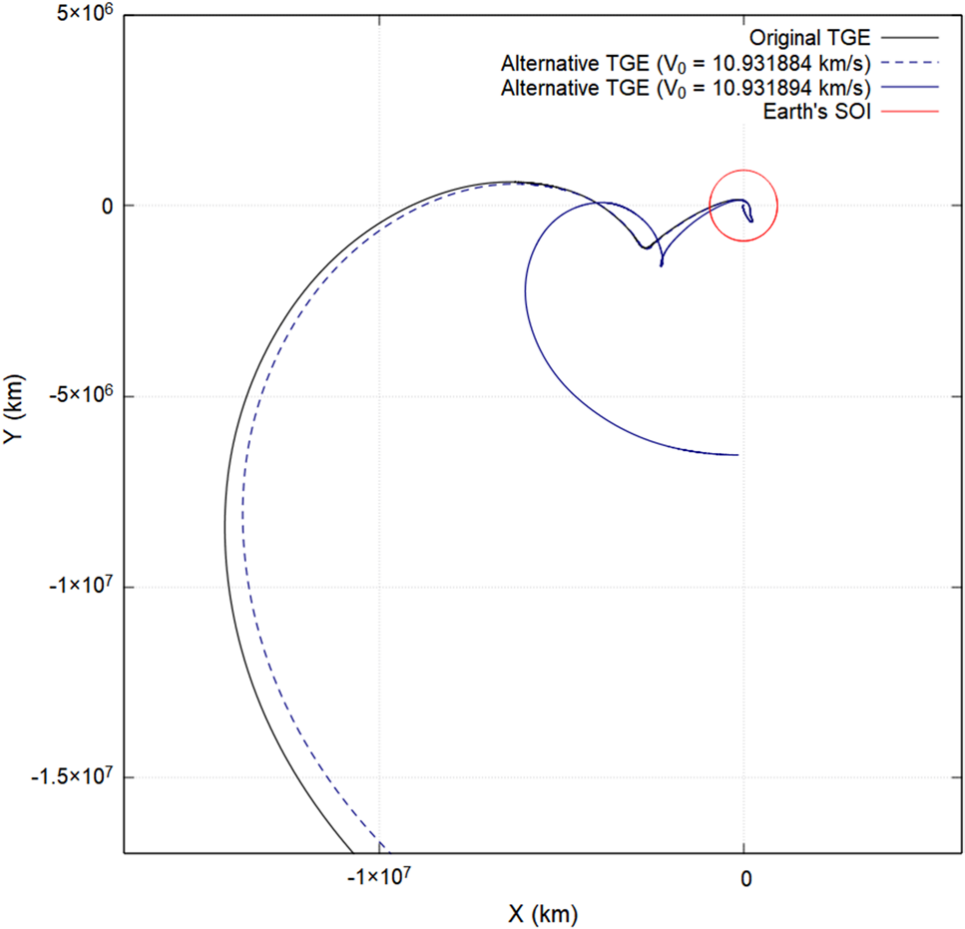
Trajectory of the proposed TGE and some of its possible deviations.

However, it is also noteworthy that one of the possible applications for the sail is the correction of the path of a TGE. As these trajectories are originated from swing-bys and this sensitive maneuver might introduce uncertainties and deviations from the intended TGE, the steering of the solar sail could easily compensate unplanned errors and return the spacecraft to the proposed trajectory. Figure 9 shows, in the geocentric reference frame, the originally proposed TGE ($V_{SC}$ = $10.931883\;{\text{km}}/{\text{s}}$), a similar TGE with a slightly different launch velocity ($V_{SC}$ = $10.931884\;{\text{km}}/{\text{s}}$), and a TGE that also has just a slightly different launch velocity ($V_{SC}$ = $10.931894\;{\text{km}}/{\text{s}}$), but a more complex path.

**Figure 10:**
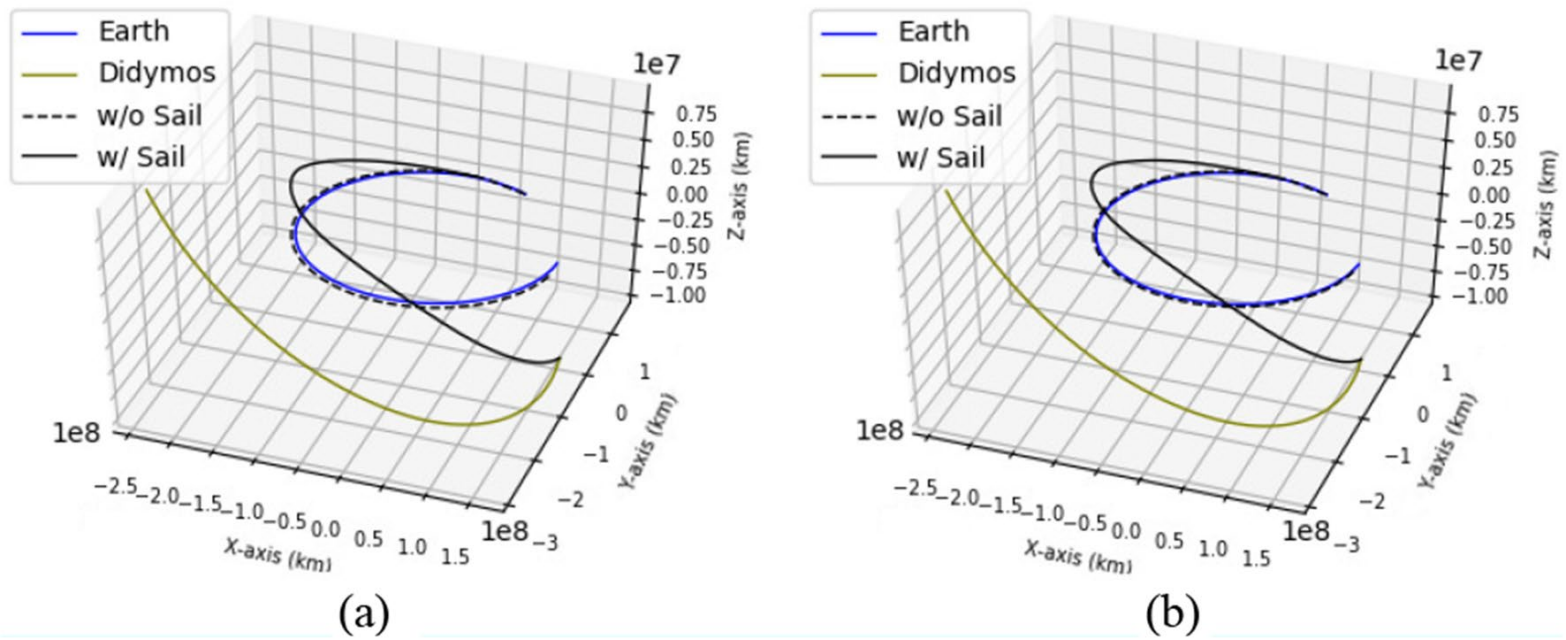
(**a**) Interception using the TGE with $V_{SC}$ = $10.931884\;{\text{km}}/{\text{s}}$. (**b**) Interception using the TGE with $V_{SC}$ = $10.931894\;{\text{km}}/{\text{s}}$.

Despite the deviations, these alternatives TGE could also be used to plan a very similar interception as the one present in Fig. 8. They have the same tof and the final values of the parameters P1, P2 and P3 are − 4.53, + 0.55, and − 0.05 m, respectively, for the first alternative trajectory and − 4.73, − 0.63, and − 0.19 m, respectively, for the second one. Figure 10 shows the interceptions conducted using these alternative trajectories.

#### Sail loading of 61.2 g/m^2^

Initially, the aim was to maintain the same launch date and tof of the DART mission, with the goal of replicating a successful mission while testing and validating the proposed technique. But this proved to be insufficient for the two larger sail loading values considered (61.2 and 164.7 g/m^2^). A larger sail loading required longer flight time so that the sail was able to guide the spacecraft into a successful collision course. This is what motivated the search for earlier launch dates and, consequently, larger times of flight. This search was conducted considering 3-month steps for the launch dates, keeping the same interception date as DART.

As previously explained, for this time frame, transfers using the other loadings were not possible, as the sail could not provide the necessary acceleration to provoke the interception, especially due to the different orbital planes of the initial TGE and Didymos, as seen in Fig. 8a. Given the low thrust nature of solar sails, a larger transfer time was needed.

To deal with this situation, new sets of dates prior to the original launch date were investigated and new TGEs were generated to determine the shortest period in which a transfer would be possible given a sail loading. For all the following alternative missions proposed, the interception date was kept at September 26, 2022.

**Figure 11:**
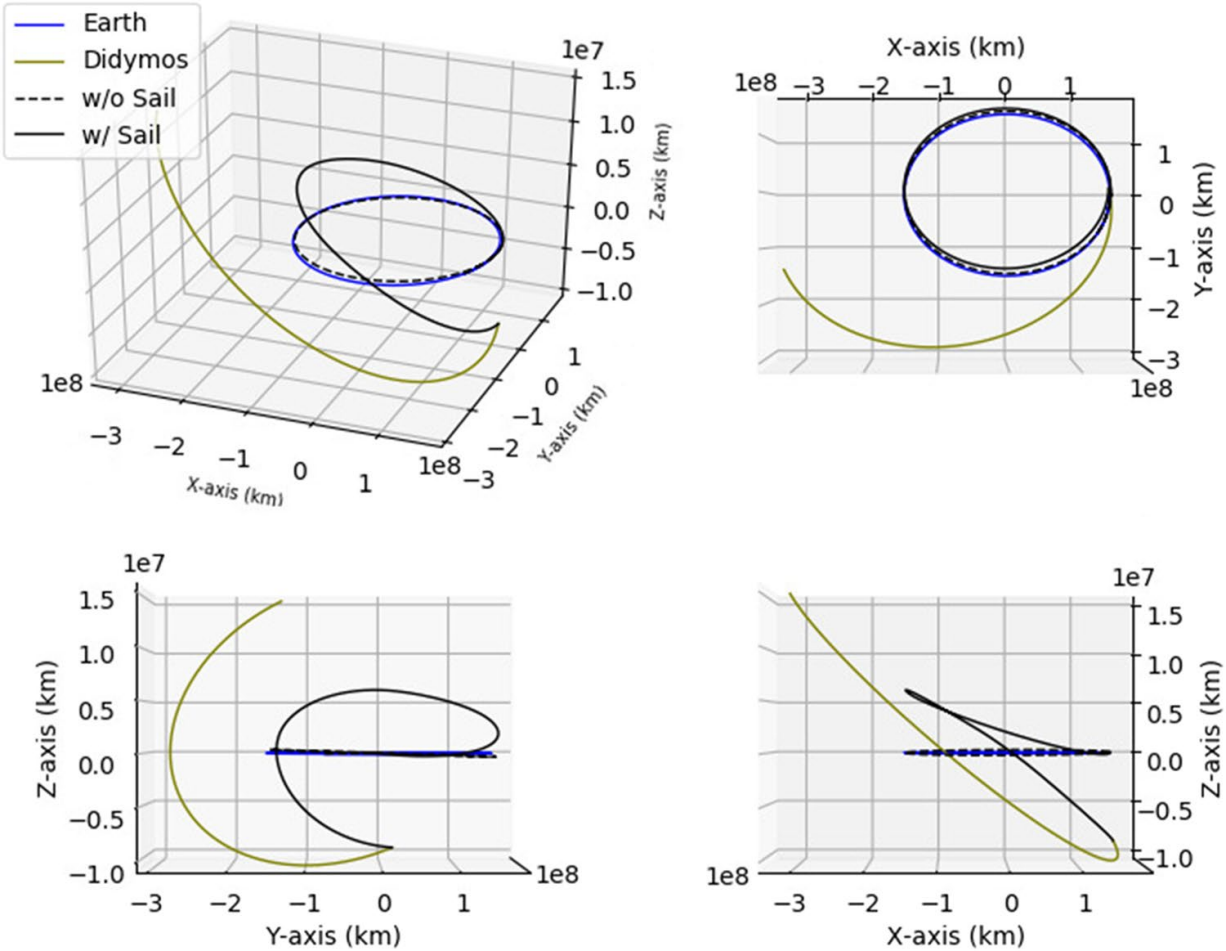
Didymos interception for a tof of 398 days and sail loading of $61.2 {\text{g}}/{\text{m}}^{2}$.

Setting the launch date to August 24, 2021, three months earlier than the original launch, a transfer with a load of $\sigma = 61.2\;{\text{g}}/{\text{m}}^{2}$ was already feasible. Figure 11 shows this transfer with a tof of 398 days from different views, in which it is possible to notice the wide change of planes necessary to accomplish the interception.

**Figure 12:**
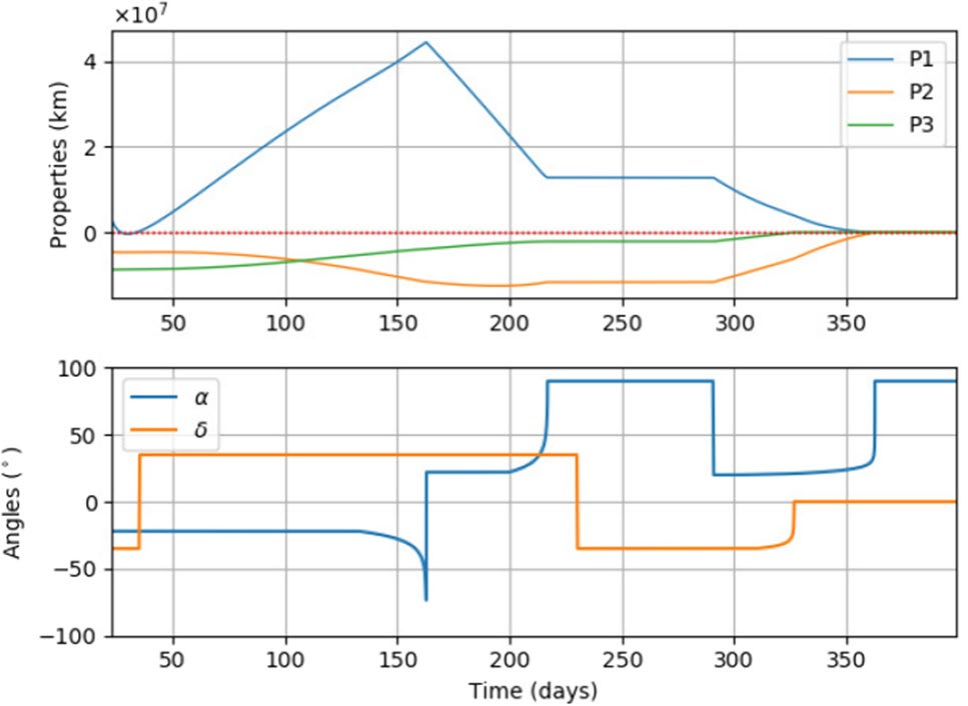
Sail properties for a tof of 398 days and sail loading of $61.2 {\text{g}}/{\text{m}}^{2}$.

As the $\Delta {V}_{L}$ needed to generate a transfer orbit with a longer period for these new intervals would be greater, henceforth all the new $\Delta V_{TGE}$ are compared with the previous $\Delta V_{L}$ of $3.434822\;{\text{km}}/{\text{s}}$. Thus, this 398 days transfer enables an 8.01% decrease in the $\Delta V$, as the initial TGE was generated with a $\Delta V_{TGE} = 3.159680\;{\text{km}}/{\text{s}}$. The evolution in time of the properties and angles can been seen in Fig. 12, the former with final values of P1 =  + 5.9 m, P2 =  + 0.3 m, and P3 = − 1.3 m.

#### Sail loading of 164.7 g/m^*2*^

For the load of $\sigma =164.7 \mathrm{g}/{\mathrm{m}}^{2}$, a larger interval was still necessary. Transfers with launch dates six, nine, twelve and fifteen months earlier were attempted with no positive outcome. The earliest launch date for which a successful interception was possible was May 24, 2020, approximately eighteen months earlier, which lead to a tof of 855 days.

**Figure 13:**
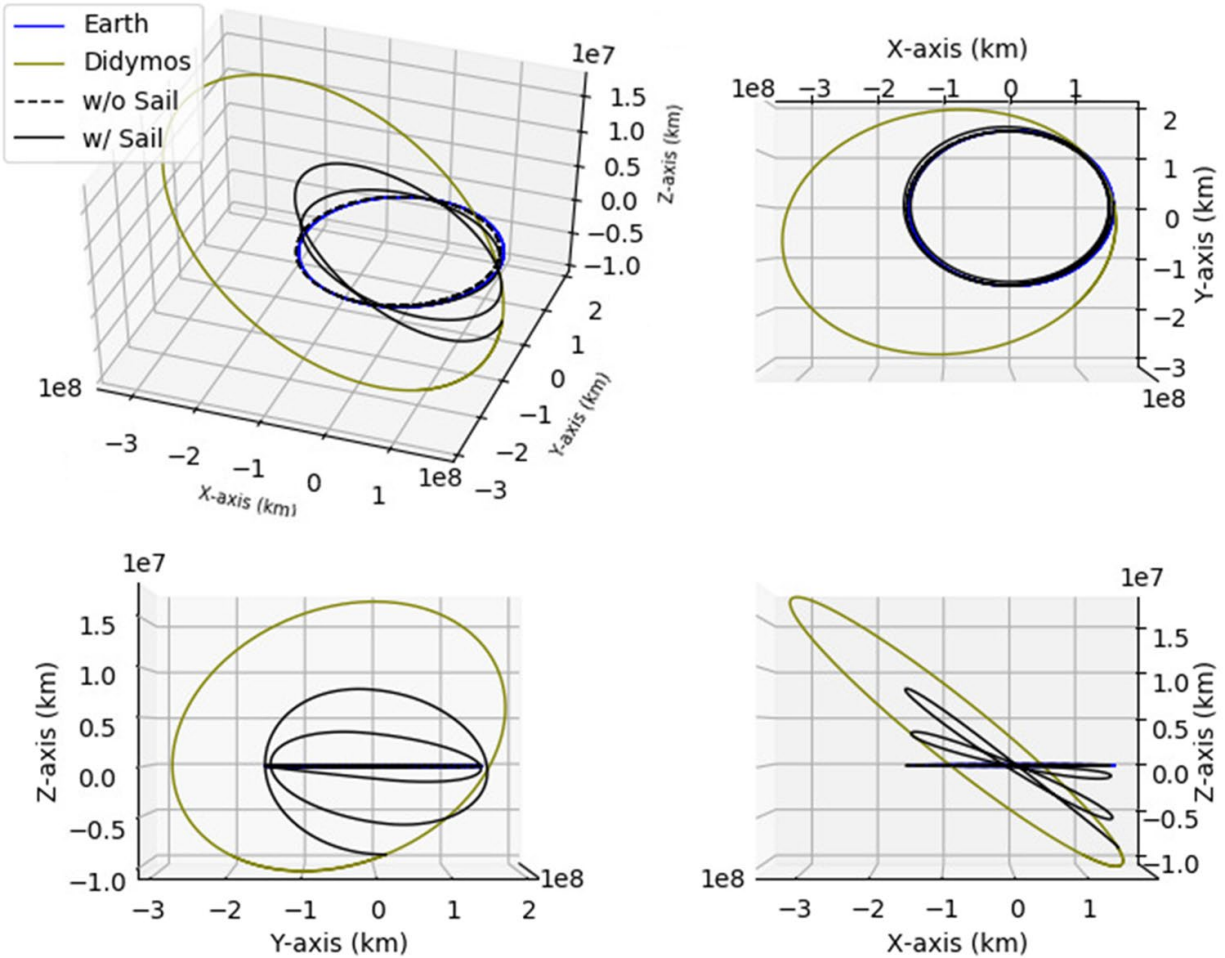
Didymos interception for a tof of 855 days and sail loading of $164.7\;{\text{g}}/{\text{m}}^{{2}}$.

**Figure 14:**
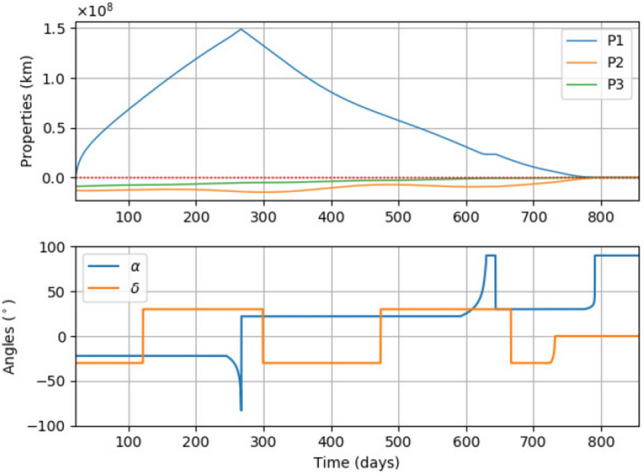
Sail properties for a tof of 855 days and sail loading of $164.7\;{\text{g}}/{\text{m}}^{{2}}$.

In Fig. 13, the spacecraft trajectory and asteroid interception are presented in different perspectives, while in Fig. 14 the sail orientation properties are shown, with the final values of P1 =  + 2.1 m, P2 = − 0.7 m, and P3 = − 0.7 m. For this case, in which $\Delta V_{TGE} = 3.157822{\text{ m}}/{\text{s}}$, the economy in the velocity increment was of 8.06%.

#### Fuel saving and further discussions

Up to this point, we conclude that this proposed hybrid mission enables savings of at least 8.0% in the $\Delta V$s. But, at the same time, it also includes more complexity to the payload structure and mass to the final stages since a solar sail is now attached to the CubeSat platform. The aim of this subsection is to discuss the benefits of this added structure.

For the purposes of this analysis some points should firstly be clarified:

- As stated in previous sections, the only $\Delta V$ considered in the mission is the $\Delta {V}_{TGE}$ applied to inject the spacecraft into a TGE from a circular LEO. The interception maneuvers are conducted only by the steering of the sail and the acceleration provided by the SRP (${a}_{SRP}$).
- As a CubeSat platform is the one envisioned for the interception mission, any propulsion module that it has would not have the required thrust to deliver $\Delta {V}_{TGE}$, so that would be up to the final launch stage selected for the mission.
- Even if the CubeSat doesn’t have a solar sail, it is reasonable to assume that it has some kind of propulsion module to perform trajectory corrections. Depending on the source of acceleration energy, propulsion systems for CubeSats could weight (dry mass) from less than 10 g to 1.5 kg and have thrusts varying from 1.0 μN to 1 N^34^. For comparison purposes, VACCO’s cold gas Micro Propulsion System (MiPS)^35^ that was used in NEA Scout mission is used as reference.

**Figure 15:**
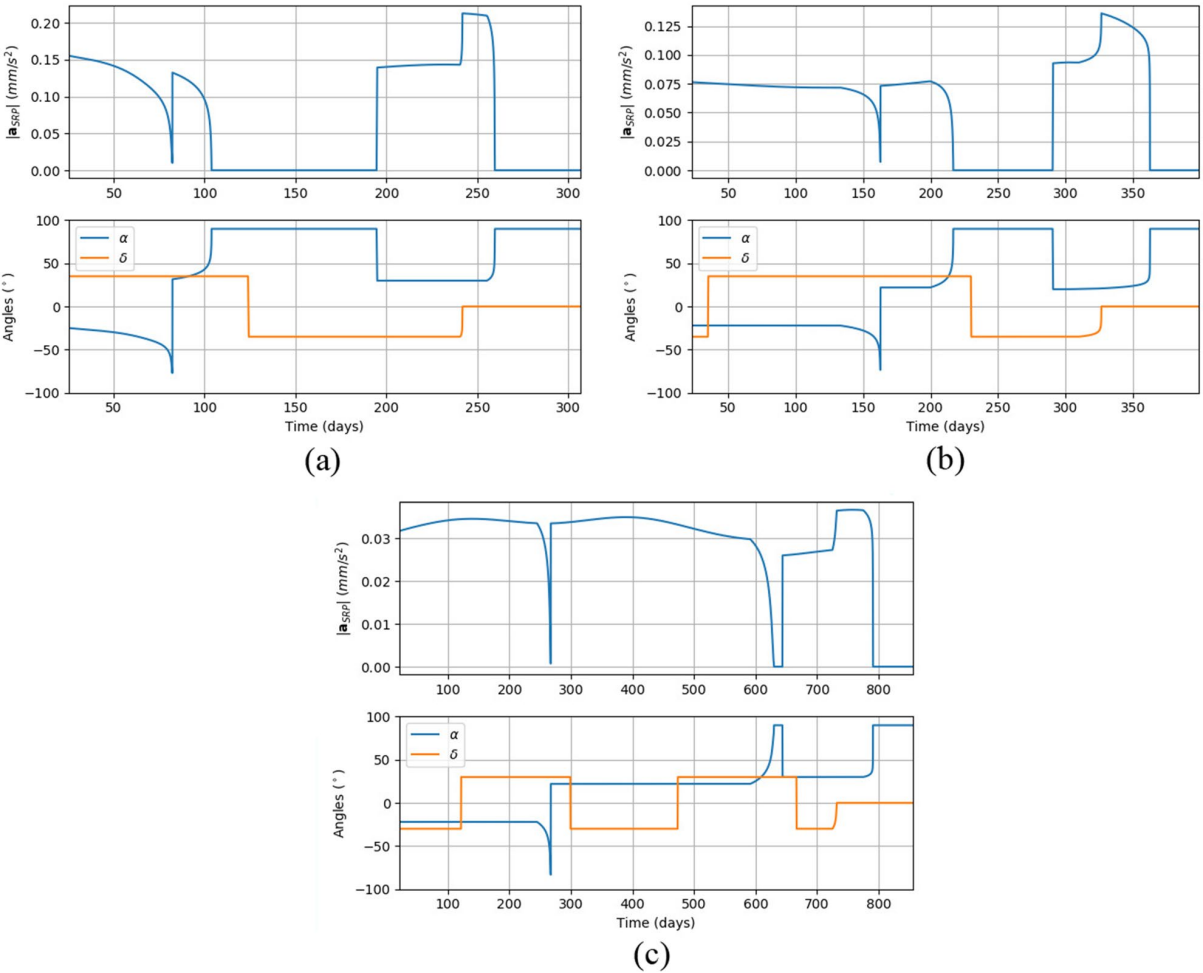
Sail steering angles and acceleration for missions with different sail loadings (**a**) $\sigma = 30.6\;{\text{g}}/{\text{m}}$. (**b**) $\sigma = 61.2\;{\text{g}}/{\text{m}}^{{2}}$. (**c**) $\sigma = 164.7\;{\text{g}}/{\text{m}}^{{2}}$.

Figure 15 shows in the upper frame the acceleration provided by the sail per mission time, while the same steering angles shown in Figs. 8, 12, and 14 are presented in the bottom frame. It is possible to notice that, if necessary, the sail can provide acceleration for the entire duration of the mission.

Multiplying the acceleration shown in Fig. 15 step by step of the integration, the total increment of velocity in each mission is 2.098 km/s ($\sigma = 164.7\;{\text{g}}/{\text{m}}^{{2}}$), 1.887 km/s ($\sigma = 61.2\;{\text{g}}/{\text{m}}^{{2}}$) and 1.730 km/s ($\sigma = 30.6\;{\text{g}}/{\text{m}}^{{2}}$). Regarding the rocket equation^32^ (Eq. 9), the maximum $\Delta V$ that MiSP could provide is 274 m/s, with its dry mass (${m}_{f}$) of 1.263 kg and wet mass (${m}_{0}$) of 2.540 kg^35^ and assumed specific impulse (${I}_{sp}$) of 40s^34^.

9 $${m}_{f}={m}_{0}{e}^{-\Delta V/({I}_{sp}{g}_{0})}$$

Thus, one of the benefits of the solar sail is acceleration unrestricted by the fuel capacity of the propulsion system. Moreover, if the CubeSat were only equipped with a conventional propulsion system such as MiSP, it would be impossible to accomplish the mission as the required $\Delta V$ wouldn’t be delivered.

Now, addressing the issue of the added mass of the solar sail, we’ll once again regard Eq. (9), where ${m}_{f}$ is now taken as

$$m_{f} = m_{s} + m_{pl} + m_{{p_{f} }} ,$$

in which ${m}_{s}$ is the mass of the launch stage structure, ${m}_{pl}$ is the mass of the payload (CubeSat including MiSP with or without solar sail), ${m}_{{p}_{f}}$ is the remaining mass of propellant after the $\Delta V$ burn.

In turn, ${m}_{0}$ is

$$m_{0} = m_{s} + m_{pl} + m_{{p_{i} }} ,$$

in which ${m}_{{p}_{i}}$ is the amount of propellant before the $\Delta V$ burn. The masses of the CubeSat (including MiSP) and solar sail are 7.98 and 6.02 kg, respectively. A ${I}_{sp}$ of 400 s, usual for upper stages, is also considered for the launch stage in the analysis of two scenarios:

- The payload doesn’t include the solar sail, i.e., ${m}_{pl}=7.98$ kg, and the launch stage performs a burn of $\Delta V=\Delta {V}_{L}= 3.434822 \mathrm{km}/\mathrm{s}$.
- The payload includes the solar sail, i.e., ${m}_{pl}=14.0$ kg, and the launch stage performs a burn of $\Delta V=\Delta {V}_{TGE}= 3.157822\mathrm{ km}/\mathrm{s}$.

**Figure 16:**
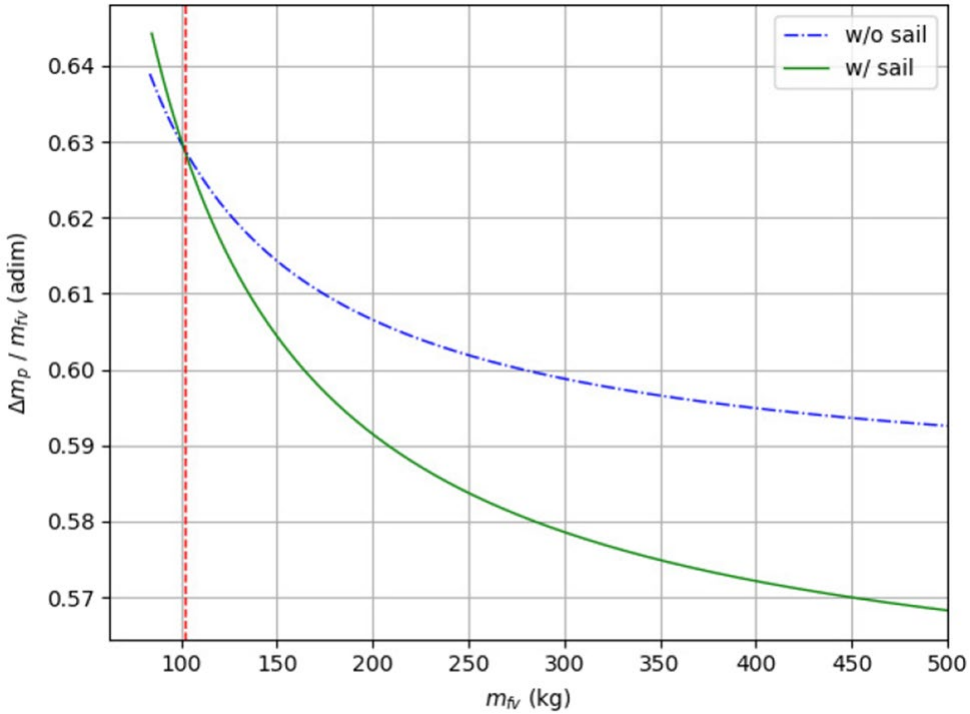
Normalized propellant mass variation per flight vehicle mass.

Figure 16 shows the fuel consumption (i.e., ${\Delta m}_{p}={m}_{0}-{m}_{f}$) normalized by the flight vehicle mass (${m}_{fv}={m}_{s}+{m}_{{p}_{i}}$) in the y-axis and the masses of the flight vehicle in the x-axis, for both scenarios.

Despite the larger mass considered in case (b) due to the addition of the solar sail, if ${m}_{fv}$ is at least 101.6 kg, the $\Delta V$ reduction compensates this added weight and there is a saving of the required propellant for the burn. It is also visible that the savings increase with ${m}_{fv}$. For example, at the 500 kg mark, the difference between the two cases is 12.2 kg.

It is noteworthy that this case, ${m}_{fv}=101.6$ kg, is the worst-case scenario, in which a dedicated launch would be the one selected for the mission and a specific launcher would be designed to meet this mission requirements. Any stage with mass greater than $101.6$ kg leads to savings in the fuel consumption.

Moreover, the usual is for CubeSats to be deployed as secondary payloads in piggyback launches. Table 1 shows some features of upper stages of different launch systems that can send payloads into escape trajectories. It is possible to notice that a stage with mass as small as 101.6 kg is hardly possible.

**Table 1:** Mass and structural coefficient for different launch stages.

Stage	Mass (kg)	Structural coefficient ($\varepsilon$)
Saturn S-IVB^36^	105,000	0.093
Delta IV-H^36^	30,710	0.11
Atlas V^37^	20,830	0.097

The structural coefficient ($\varepsilon$) is defined by Eq. (10)^36^. Although theoretically it would be possible to build small stages with ${m}_{s}=2 \mathrm{kg}$ and ${m}_{{p}_{i}}=18$ kg, for the typical value of $\varepsilon =0.1$ seen in Table 1, in practice this small stage wouldn’t meet the propulsion requirements of most missions.

10 $$\varepsilon =\frac{{m}_{s}}{{m}_{s}+{m}_{{p}_{i}}}$$

For example, even Altair, the fourth stage of the Scout Lauch Vehicle Program^38^, that is among the smallest upper stages ever flown, surpass this threshold with a structural coefficient of 0.15, dry mass of 41 kg and total mass of 275 kg. In conclusion, in more realistic launch scenarios, the $\Delta V$ reduction could lead to great savings in the propellant burn.

## Conclusions

This work presents a new methodology to generate low-cost trajectories towards Near-Earth Objects by the combined use of escape trajectory from the Earth-Moon system (Trajectories G or TGE) and solar sails. Among the benefits brought by the use of this methodology are the capability to reorient the spacecraft in order to reach a predetermined target, and the compensation of small deviations without the expenditure of additional propellant.

In the Restricted Four-Body Sun-Earth-Moon-spacecraft Problem, this strategy provided an increase on the savings of the required $\Delta V$ from 4.89 to 6.92%, by increasing the vehicle reach mwithout using new speed increments. A direct consequence of the savings in the $\Delta V$ are savings in the mass of propellant required to perform the maneuvers. This freed weight that could be used to allocate more equipment or payloads.

The heuristic techinique was applied to plan an interception mission to the NEA 65803 Didymos, with the conclusion that with the current solar sail technology, reflected in the sail loadings $\left( \sigma \right)$, and restricted time frame of the mission, it would not be possible to perform the transfer with the methodology proposed. However, given more time or using solar sails with smaller loadings, the economy on the $\Delta V_{{\text{s}}}$ required is up to 8.48% when compared to Lambert’s method.

Considering a load of $\sigma =30.6$ g/m^2^, an interception with the same time frame of the DART mission is possible, with the bonus of an 8.48% reduction in the $\Delta V$ required by the mission. For a $\sigma =61.4$ g/m^2^, an earlier launch of three months is required for the success of the mission, providing an 8.01% reduction. The sail loading of $\sigma =164.7$ g/m^2^, similar to the ones used in recently launched missions, needed the larger time of flight to be successfully performed, 855 days, but still lead to an 8.06% reduction on the velocity increments.

## Data Availability

The datasets used and/or analyzed during the current study available from the corresponding author on reasonable request.

## References

[1] Grier J, Rivkin AS (2018). Airless Bodies of the Inner Solar System: Understanding the Process Affecting Rocky, Airless Surfaces.

[2] National Academies of Sciences, Engineering and Medicine. Origins, Worlds, and Life: A Decadal Strategy for Planetary Science and Astrobiology 2023–2032 (2022).

[3] Bignami G, Cargill P, Schutz B, Turon C (2005). Cosmic Vision: Space Science for Europe 2015–2025.

[4] NASA. NASA, SpaceX Launch DART: First Test Mission to Defend Planet Earth. https://www.nasa.gov/press-release/nasa-spacex-launch-dart-first-test-mission-to-defend-planet-earth (2021).

[5] NASA. NASA Confirms DART Mission Impact Changed Asteroid’s Motion in Space. https://www.nasa.gov/press-release/nasa-confirms-dart-mission-impact-changed-asteroid-s-motion-in-space (2022).

[6] Michel P (2022). The ESA Hera mission: Detailed characterization of the DART impact outcome and of the binary asteroid (65803) Didymos. Planet Sci. J..

[7] Jet Propulsion Laboratory. NEA Scout. https://www.jpl.nasa.gov/missions/near-earth-asteroid-scout-neascout (2021).

[8] Johnson, L. *et al.* Near Earth Asteroid Scout Mission Update (2020).

[9] Jet Propulsion Laboratory. Near-Earth Object Surveyor. https://www.jpl.nasa.gov/missions/near-earth-object-surveyor (2022).

[10] Randolph, T. M. NEO Surveyor (2011).

[11] Farquhar RW (2001). The flight of ISEE-3/ICE: Origins, mission history, and a legacy. J. Astronaut Sci..

[12] Lawden DF (1954). Perturbation maneuvers. J. Br. Interplanet. Soc..

[13] Minovitch M (1961). A method for determining interplanetary free-fall reconnaissance trajectories. JPL Tec. Memo.

[14] Broucke, R. The celestial mechanics of gravity assist. In *Astrodynamics Conference* (1988).

[15] Negri RB, Prado AF (2020). A historical review of the theory of gravity-assists in the pre-spaceflight era. J. Braz. Soc. Mech. Sci. Eng..

[16] Broucke, R. A. Periodic orbits in the restricted three body problem with earth-moon masses (1968).

[17] De Melo CF, Winter OC (2006). Alternative paths to Earth-Moon transfer. Math. Probl. Eng..

[18] De Melo CF, Macau EEN, Winter OC (2009). Alternative transfers to the neos 99942 apophis, 1994 wr12, and 2007 uw1 via derived trajectories from periodic orbits of family g. Math. Probl. Eng..

[19] de Melo CF, Macau EEN, Winter OC (2009). Strategies for plane change of Earth orbits using lunar gravity and derived trajectories of family G. Celest. Mech. Dyn. Astron..

[20] Ribeiro RS, de Melo CF, Prado AF (2022). trajectories derived from periodic orbits around the Lagrangian point L1 and lunar swing-bys: Application in transfers to near-earth asteroids. Symmetry.

[21] Frisbee RH (2003). Advanced space propulsion for the 21st century. J. Propuls. Power.

[22] Gong S, Macdonald M (2019). Review on solar sail technology. Astrodynamics.

[23] Spencer DA (2021). The LightSail 2 solar sailing technology demonstration. Adv. Space Res..

[24] Howell, E. NASA’s Artemis 1 mission launched 10 cubesats. Here’s how they’re doing. *Space.com* https://www.space.com/artemis-1-cubesats-deep-space-updates (2022).

[25] Porter, M. & O’Neill, I. J. NEA Scout Status Update. *NASA Website* https://www.nasa.gov/centers/marshall/news/2022/nea-scout-status-update.html (2022).

[26] Murray CD, Dermott SF (1999). Solar System Dynamics.

[27] Vulpetti, G., Johnson, L. & Matloff, G. L. Solar Sails. Preprint at (2008).

[28] Ceriotti, M., McInnes, C. R. & Diedrich, B. L. The pole-sitter mission concept: An overview of recent developments and possible future applications. In *62nd International Astronautical Congress 2011* (2011).

[29] Meireles LG, Prado AF, de Melo CF, Pereira MC (2023). Heuristic technique for the search of interception trajectories to asteroids with the use of solar sails. Symmetry.

[30] Peloni A, Ceriotti M, Dachwald B (2016). Solar-sail trajectory design for a multiple near-earth-asteroid rendezvous mission. J. Guid. Control Dyn..

[31] Mengali G, Quarta AA (2005). Optimal three-dimensional interplanetary rendezvous using non-ideal solar sail. J. Guid. Control Dyn..

[32] Curtis HD (2009). Orbital Mechanics for Engineering Students.

[33] Jet Propulsion Laboratory (JPL). Horizons System. https://ssd.jpl.nasa.gov/horizons/

[34] Lemmer K (2017). Propulsion for cubesats. Acta Astronaut.

[35] VACCO Industries. NEA Scout Propulsion System. *VACCO Cubesat Propulsion Systems*. https://cubesat-propulsion.com/nea-scout-propulsion-system/

[36] Griffin, M. D. *Space vehicle design* (AIAA, 2004).

[37] Alliance, U. L. Atlas V Launch services user’s guide. *Lockheed Martin Commercial Launch Services* (2010).

[38] Tanck, P. & Williams, J. The Scout launch vehicle system (1988).

